# Expression, tissue localization and serodiagnostic potential of *Taenia multiceps* acidic ribosomal protein P2

**DOI:** 10.1186/s13071-015-1220-8

**Published:** 2015-12-01

**Authors:** Xing Huang, Lin Chen, Yingdong Yang, Xiaobin Gu, Yu Wang, Weimin Lai, Xuerong Peng, Guangyou Yang

**Affiliations:** Department of Parasitology, College of Veterinary Medicine, Sichuan Agricultural University, Chengdu, 611130 China; Chengdu Agricultural College, Chengdu, 611130 China; Panzhihua Animal Science and Technology Institute, Panzhihua, 617061 China; College of Science, Sichuan Agricultural University, Ya’an, 625014 China

**Keywords:** *Taenia multiceps*, Acidic ribosomal protein P2, Immunofluorescence, Indirect ELISA

## Abstract

**Background:**

The larval stage of *Taenia multiceps*, also known as coenurus, is the causative agent of coenurosis, which results in severe health problems in sheep, goats, cattle and other animals that negatively impact on animal husbandry. There is no reliable method to identify coenurus infected goats in the early period of infection.

**Methods:**

We identified a full-length cDNA that encodes acidic ribosomal protein P2 from the transcriptome of *T. multiceps* (*Tm*P2)*.* Following cloning, sequencing and structural analyses were performed using bioinformatics tools. Recombinant *Tm*P2 (r*Tm*P2) was prokaryotically expressed and then used to test immunoreactivity and immunogenicity in immunoblotting assays. The native proteins in adult stage and coenurus were located via immunofluorescence assays, while the potential of r*Tm*P2 for indirect ELISA-based serodiagnostics was assessed using native goat sera. In addition, 20 goats were randomly divided into a drug treatment group and a control group. Each goat was orally given mature, viable *T. multiceps* eggs. The drug treatment group was given 10 % praziquantel by intramuscular injection 45 days post-infection (p.i), and all goats were screened for anti-*Tm*P2 antibodies with the indirect ELISA method established here, once a week for 17 weeks p.i.

**Results:**

The open reading frame (366 bp) of the target gene encodes a 12.62 kDa protein, which showed high homology to that from *Taenia solium* (93 % identity) and lacked a signal peptide. Immunofluorescence staining showed that *Tm*P2 was highly localized to the parenchymatous zone of both the adult parasite and the coenurus; besides, it was widely distributed in cystic wall of coenurus. Building on good immunogenic properties, r*Tm*P2-based ELISA exhibited a sensitivity of 95.0 % (19/20) and a specificity of 96.3 % (26/27) in detecting anti-P2 antibodies in the sera of naturally infected goats and sheep. In goats experimentally infected with *T. multiceps*, anti-*Tm*P2 antibody was detectable in the control group from 3 to 10 weeks and 15 to 17 weeks p.i. In the drug-treated group, the anti-*Tm*P2 antibody dropped below the cut-off value about 2 weeks after treatment with praziquantel and remained below this critical value until the end of the experiment.

**Conclusion:**

The indirect ELISA method developed in this study has the potential for detection of *T. multiceps* infections in hosts.

## Background

*Taenia multiceps* is a widespread parasite in many areas of the world. The larval stage, known as coenurus, mainly parasitizes the brain or spinal cord of domestic ruminants such as buffalo, cattle, goats, sheep, and yak, as well as wild species, causing lethal neurological symptoms [1–4]. Adult *T. multiceps* inhabit the small intestine of dogs, wolves, foxes and other canids [1–4]. *T. multiceps* induced coenurosis occurs almost all over the world [5], causing enormous economic losses to the livestock industry and threatening human health [1–5].

The rapid diagnosis of coenurus infection in hosts is crucial to control coenurosis and reduce its negative impact on animal husbandry. However, because *T. multiceps* infections in goats do not cause obvious early clinical symptoms, it is a significant challenge to diagnose the disease in the early stage. In recent decades, as molecular biological understanding of parasites has increased, many researchers have screened recombinant antigens for diagnosis of diseases caused by the family Taeniidae (which includes many tapeworms of medical and veterinary importance). However, data are limited on recombinant diagnostic antigens for coenurus [6–13]. Although different methods such as enzyme-linked immunosorbent assay (ELISA) [14], dot immunogold filtration assay (DIGFA) [15] and Dot-ELISA [16] have been developed for the diagnosis of coenurosis, these assays use natural worm extracts as antigens and therefore cannot be produced as commercial products. When compared with ELISA based on natural worm antigens, indirect ELISA using recombinant proteins as the capture antigen has many advantages, including high reproducibility and a stable antigen source.

Acidic ribosomal proteins are so named because of their acidic isoelectric point (pH = 3–5) and their origin in the prokaryotic or eukaryotic ribosomal large subunit. They play important roles in the maintenance of stability and activity of the ribosome [17–20], by interacting with the elongation factor involved in the regulation of protein synthesis [21–23]. Moreover, several studies have confirmed that the acidic ribosomal proteins of eukaryotes play a role in apoptosis [24, 25], the occurrence and invasion of tumors [26–28] and immune diseases [29, 30]. Acidic ribosomal proteins of eukaryotic cells are divided into three types, P0, P1 and P2 (collectively called P-proteins). The P-proteins form the lateral stalk complex of the large ribosomal subunit, comprising a 32 to 35 kDa P0 protein at the core, to which two heterodimers of acidic ribosomal proteins P1 and P2 (about 12–14 kDa each) bind, ultimately forming the stalk P0-(P1/P2)_2_ complex [31]. Martinez-Azorin F et al. (2008) [32] research showed that P1/P2 proteins in human cells modulate cytoplasmic translation by influencing the interaction between subunits, thereby regulating the rate of cell proliferation the recombinant P2 proteins.

The aim of this study was to characterize the *Tm*P2 protein of *T. multicelps*, determine its tissue distribution and further develop an indirect ELISA assay for the serodiagnosis of coenurosis by using recombinant *Tm*P2.

## Methods

### Ethics statement

All animals were raised strictly according to the animal protection laws of the People's Republic of China (a draft of an animal protection law released on September 18, 2009). All procedures were carried out in strict accordance with the Guide for the Care and Use of Laboratory Animals by the Animal Ethics Committee of Sichuan Agricultural University (Ya’an, China) (Approval No. 2012–030).

### Animals

Two healthy New Zealand white rabbits were obtained from the Laboratory Animal Center of Sichuan Agricultural University. Twenty healthy goats were obtained from a goat farm in Sichuan Province, China. All animals were fed in a barrier environment and provided with food and clean water *ad libitum*. Animals were adapted to the new environment for 1 week before the experiment.

### Parasites

Adult *T. multiceps* derived from artificially infected dogs were provided by the Department of Parasitology, College of Veterinary Medicine, Sichuan Agricultural University. Coenuri were isolated from the brain of naturally infected goats in a goat farm in Sichuan Province. All materials were stored in liquid nitrogen until use.

### Cloning, expression and purification of recombinant *Tm*P2

The total RNA of coenurus was extracted using a commercial kit (Huashun, Shanghai, China) and cDNA was transcribed using a RevertAid™ First Strand cDNA Synthesis Kit (MBI Fermentas, Germany) according to the manufacturers’ protocols and stored at −70 °C. Based on the transcriptome data of *T. multiceps* and the P2 sequence of *T. solium* (GenBank: L39653), gene-specific primers for *Tm*P2 were designed as follows: F1 5ʹ-CCGGAATTCATGCGCTATCTCGCTGCTTAT-3ʹ and R1 5ʹ-CGGCTCGAGTTAGTCAAAGAGACTGAAACCCAT-3ʹ (Invitrogen, Carlsbad, USA), which incorporated *Eco*RI and *Xho*I restriction sites, respectively. The PCR products were digested and gel-purified (Novagen, Madison, Germany). The cDNA was subcloned into the bacterial expression vector pET-32a(+) (Novagen) and used to transform *Escherichia coli* BL21 (DE3) cells (Novagen). *E. coli* cells were cultivated at 37 °C, and then induced by Isopropyl − β − D-thiogalactopyranoside (IPTG) at an ultimate concentration of 1 mM. The purity of the expressed protein was measured as previously described [33].

### Sequence analysis

The presence of a signal peptide was assessed using SignaIP 4.1 at the Center for Biological Sequence Analysis website (http://www.cbs.dtu.dk/services/SignalP/), and cellular localization was predicted using TMHMM (http://www.cbs.dtu.dk/services/TMHMM/). The molecular weight and pI values of the predicted protein were calculated using Compute pI/Mw at ExPasy (http://web.expasy.org/protparam/).

### Sera

Positive sera against parasites coenurus (20 samples) and C*ysticercus tenuicollis* (7 samples) were isolated from naturally infected goats from a goat farm in Sichuan Province, and *Echinococcus granulosus* (8 samples) isolated from naturally infected sheep. Negative sera (24 samples) were collected from 24 cestode-free goats by autopsy. All sera were stored at −20 °C until use.

### Western blot analysis

Protein extracts were prepared by homogenizing coenurus in an NP-40 cell lysis buffer (Boster, Wuhan, China). Purified r*TmP2* proteins and total worm extract were separated by SDS-PAGE and transferred onto Polyvinylidene Fluoride membranes (Boster) for 30 min in an electrophoretic transfer cell (Bio-Rad, USA). The membrane was blocked with 5 % skim milk in Tris Buffered Saline with Tween-20 (TBST) for 2 h at room temperature. Membranes were then incubated overnight at 4 °C with goat antiserum from naturally infected goats (diluted 1:100 (v/v) in 1 % skim milk in TBST). And the rest of the program was performed as described previously [34].

### Immunofluorescence

To perform immunolocalization studies, *T. multiceps* sections were probed with specific rabbit anti-*Tm*P2 antibodies (1:300) followed by fluorescein isothiocyanate (FITC)-conjugated goat anti-rabbit IgG (1:200; Boster, Wuhan, China) as described elsewhere [34]. The stained samples were mounted in glycerol/phosphate buffer (v/v, 9:1) and viewed under an Olympus BX50 fluorescence microscope (Olympus, Japan). Negative controls were prepared using uninfected goat serum instead of specific antibodies.

### Development of the indirect ELISA

The optimal concentration of ELISA reagents (*Tm*P2 protein and serum) was determined through standard checkerboard titration procedures [35]. Briefly, ELISAs were performed in polystyrene 96-well microtiter plates (Invitrogen) using 100 μL reaction mixtures with *Tm*P2 protein, coated at six different concentrations (0.06, 0.12, 0.24, 0.48, 0.96, and 1.92 μg/mL) in 0.1 M carbonate buffer (pH 9.6) and incubated overnight at 4 °C. After washing three times with 0.01 M PBST, the plate was blocked with 100 μl/well of 5 % skimmed milk (skimmed milk in PBS) for 2 h at 37 °C. A serial two-fold dilutions (100 μL; ranging from 1:5, 1:10, 1:20, 1:40, 1:80) of the positive and negative sera samples were used in the following step, and positive sera and negative sera were diluted in PBS. Then, after washing three times, 100 μL of HRP-labeled rabbit anti-goat IgG diluted 1:2000 in 0.01 M PBST were added to each well, followed by a 1 h incubation at 37 °C and washing three times. Finally, 100 μL tetramethylbenzidine were added into every well, and incubated at 37 °C for 20 min in the dark. After the reaction was stopped, we determined the absorbance at 450 nm in an automatic ELISA plate reader. The conditions which gave the highest P/N value and an OD450 value for positive serum close to 1.0 were defined as the optimal working conditions [36].

### Determination of the cut-off value for the indirect ELISA

Twenty-four samples of coenurus negative sera were used to assess the cut-off value under the optimal conditions for indirect ELISA. The cut-off value was calculated as the mean OD450 plus three standard deviations (SD) and will be used as a standard to identify sera positive and negative for coenurus.

### Sensitivity and specificity analysis of indirect ELISA

The percentage sensitivity was calculated as indirect ELISA positive × 100/true positive, and the percentage specificity was calculated as indirect ELISA negative × 100/true negative. The specificity was evaluated by cross-reaction with antibody derived from *E. granulosus*-positive sheep and *C. tenuicollis*-positive goats.

### Detection of anti-*Tm*P2 antibody in goats infected with *T. multiceps* by ELISA

Twenty healthy adult goats were randomized to a drug treatment group and a control group (10 in each group). Each goat was orally given an average of 5500 mature, viable *T. multiceps* eggs. At 45 post infection (p.i.), the drug treatment group was given 10 % (w/v) praziquantel by intramuscular injection at a dose of 70 mg/kg of body weight, once each day. Serum samples were collected from all the goats at weekly intervals until 17 weeks p.i.

## Results

### Sequence analysis, expression and reactivity of r*Tm*P2

**Fig. 1:**
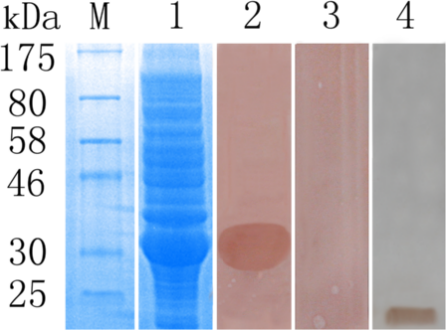
SDS-PAGE and Western blotting analysis of recombinant *Tm*P2 protein. M, Protein molecular weight markers; lane 1, the extracts of *E. coli* cells containing the pET-32a (+) expression vector with IPTG induction; lanes 2–3, r*Tm*P2 protein probed with goat immune serum against *T. multiceps* (positive serum group; lane 2) or naïve goat serum (negative control, lane 3); lane 4, the total worm extract probed with goat immune serum against *T. multiceps*

The *Tm*P2 cDNA sequence consisted of an open reading frame of 366 bp encoding a putative protein with 121 amino acid residues. The protein was predicted to have a molecular weight of 12.62 kDa, a pI of 5.01, and weak hydropathicity. No signal peptides or transmembrane domains were predicted in this protein. The protein sequence of *Tm*P2 was found to be highly homologous to those from *T. solium* (93 % identity), *Echinococcus granulosus* (81 %) and *Hymenolepis microstoma* (65 %), and it exhibited homology to the acidic ribosomal protein P2 from other parasites such as *Spirometra erinaceieuropaei*, *Barentsia elongata* and *Caenorhabditis briggsae* (51 %, 49 % and 47 % identity, respectively). Recombinant *Tm*P2 was expressed as soluble protein with a molecular weight of approximately 32 kDa (Fig. 1). Due to an additional 20-kDa epitope tag fusion peptide, the molecular mass of *Tm*P2 was ~12 kDa, similar to that predicted from its amino acid sequence. In Western blot analysis, a positive band of 32 kDa was observed when using goat anti-*T. multiceps* serum, suggesting a strong reactivity of this recombinant protein (Fig. 1). No signal was present when r*Tm*P2 was incubated with native sera (Fig. 1). In addition, the total worm extract was blotted with anti-r*Tm*P2 rabbit serum of approximately 12 kDa (Fig. 1).

### Immunolocalization of P2 protein in *T. multiceps*

**Fig. 2:**
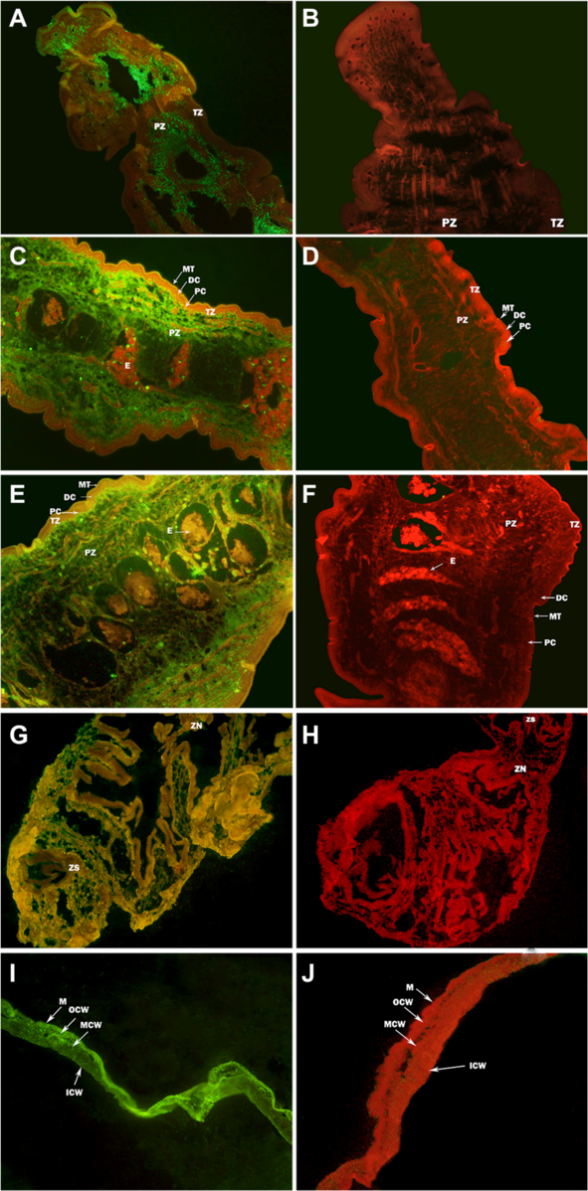
Immunolocalization of P2 protein in *T. multiceps*. The green fluorescence tint shows the location of *Tm*P2 protein. **a**, antisera in cephalomeres of adult tapeworm; **b**, negative sera in cephalomeres of adult tapeworm; **c**, antisera in immature segments of adult tapeworm; **d**, negative sera in immature segments of adult tapeworm; **e**, antisera in mature segments of adult tapeworm; **f**, negative sera in mature segments of adult tapeworm. **g**, antisera in cephalomeres of coenurus; **h**, negative sera in cephalomeres of coenurus; **i**, antisera in cystic wall of coenurus; **j**, negative sera in cystic wall of coenurus. The magnification of all images is × 200. Arrows indicate the areas of the parasite: MT, microthrix; DC, distal cytoplasm; PC, perinuclear cytoplasm; TZ, tegument zone; PZ, parenchymatous zone; E, eggs; ZS, zone of scole; ZN, zone of neck; M, microtrichia; OCW, the outer layer of cystic wall; MCW, the middle layer of cystic wall; ICW, the inside layer of cystic wall

Protein P2 was highly localized to the parenchymatous zone (PZ) of both the adult parasite and the coenurus; furthermore, it was widely distributed in cystic wall of coenurus (Fig. 2). No fluorescence staining was detected with negative sera.

### Establishment of indirect ELISA

The suitability of the recombinant *Tm*P2 protein as a diagnostic antigen was tested based on indirect ELISA. Checkerboard titration tests indicated that the conditions that gave a highest P/N value (3.64) were when the coating antigen was 0.24 μg/well and the serum dilution was 1:10 (Table 1). In the optimized test conditions, a total of 24 negative serum samples were analyzed to obtain the cut-off value for the indirect ELISA, and OD450 value was 0.312 with a SD of 0.0622 (data not shown). All experiments were performed in triplicate. Thus, the cut-off value was 0.499 (mean + 3SD). Serum samples with an absorbance ≥0.499 were scored as coenurus antibody positive, otherwise they were determined to be coenurus antibody negative.

**Table 1:** Determination of the coating concentration of protein and serum dilution in optimization of the indirect ELISA method

Antisera at different dilutions	OD450 values of antigen at different coating concentrations					
	0.06 μg	0.12 μg	0.24 μg	0.48 μg	0.96 μg	1.92 μg
1:5 (P)	1.181	1.137	1.065	1.078	1.182	1.178
1:5 (N)	0.427	0.361	0.319	0.307	0.478	0.487
1:10 (P)	0.932	0.908	0.896	0.789	0.858	0.873
1:10 (N)	0.357	0.288	0.246	0.238	0.357	0.371
1:20 (P)	0.664	0.621	0.542	0.501	0.645	0.626
1:20 (N)	0.238	0.244	0.191	0.180	0.275	0.245
1:40 (P)	0.461	0.447	0.347	0.397	0.473	0.445
1:40 (N)	0.198	0.165	0.131	0.148	0.223	0.227
1:80 (P)	0.348	0.339	0.298	0.305	0.392	0.381
1:80 (N)	0.157	0.143	0.115	0.122	0.134	0.139

Note: N, positive serum; P, negative serum; The values in bold represent optimum conditions of this indirect ELISA method

### Sensitivity and specificity analysis of indirect ELISA

**Fig. 3:**
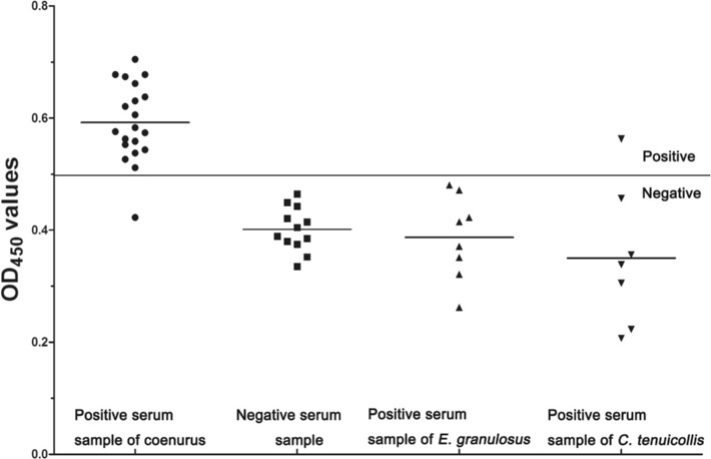
The sensitivity, specificity and cross-reactivity of indirect ELISA. The bold horizontal line indicates the cut-off value (0.499)

Specific IgG antibodies were determined in serum samples from goats infected with coenurus and *C. tenuicollis*, and from sheep infected with *E. granulosus*. (Fig. 3). Based on the cut-off of 0.499, a total of 20 serum samples from goats infected with coenurus were detected as positive, corresponding to a sensitivity of 95.0 % (19/20). There was cross-reactivity with serum from one C*. tenuicollis*-positive goat (*n* = 7) and no reactions with sera from *E. granulosus*-positive sheep (*n* = 8) and healthy goats (*n* = 12). Thus, the specificity of the ELISA using recombinant *Tm*P2 antigen to detect anti-P2 antibody was 96.3 % (26/27).

### Anti-*TmP2* antibody in goats artificially infected with *T. multiceps*

**Fig. 4:**
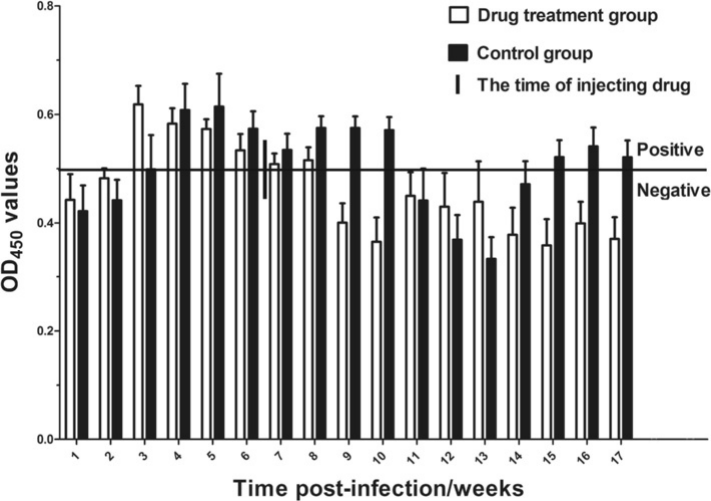
The regularities of serological antibody against *Tm*P2 after the artificial infection of goats by *T. multiceps.* The bold horizontal line indicates the cut-off value (0.499)

The regularities of serological antibodies are shown in Fig. 4 after the artificial infection of goats with *T. multiceps*. The following trends were observed: 3 weeks p.i., the drug treatment group and the control group were both positive for serum antibody to *Tm*P2 (OD value >0.499, the cut-off value). At around 9 weeks p.i. (about 2 weeks post-injection of praziquantel), the antibody value for the drug-treated group dropped below the cut-off value; the antibody values remained below this critical value until the end of the experiment. In the control group, the antibody value dropped below the cut-off value 11 weeks p.i., but the value rose again and was detection positive from 15 weeks p.i. until the end of the experiment (17 weeks p.i.).

## Discussion

In recent years, studies concerning acidic ribosomal P2 proteins of parasites have mainly focused on *Trypanosoma cruzi* [37–39], *Cryptosporidium parvum* [29, 40], *Toxoplasma gondii* and *Plasmodium falciparum* [41, 42]. These studies confirmed that P2 protein could induce hosts to produce a strong humoral immune response, and the protein appears to constitute a potential target for host cell invasion inhibition in both *T. gondii* and *P. falciparum* infections [29, 41, 42]. Knowledge of P2 protein is very limited for the family Taeniidae, although there are few preliminary studies on *T. solium* [43–45] which confirmed that P2 is a main pathogenic factor of human cysticercosis, and demonstrated that a P2 fusion protein expressed in *E. coli* could be used as a diagnostic antigen for human neurocysticercosis [43]. In addition, Luo et al. (2003) [44] and Su et al. (2003) [45] showed that the recombinant P2 proteins of *T. solium* expressed in *E. coli* and *Pichia pastoris* were good immunogens. In the present study, we have cloned and expressed *Tm*P2 from coenurus for the first time.

Immunofluorescence staining showed that, in *P. falciparum*, ribosomal protein P2 (*PfP2)* was present at the infected erythrocyte surface at the onset of cell division, and on the merozoite surface during the *P. falciparum* host infection process [41]. In the process of *T. gondii* host cell infection, P2 protein was confirmed to be localized at the surface and in the cytoplasm of the parasite [41]. In the present study, we observed that *Tm*P2 was highly localized to the parenchymatous zone of both the adult parasite and the coenurus; besides, it was widely distributed in cystic wall of coenurus, commensurate with its ribosomal role. Whether P2 is present in the body of *T. multiceps* oncospheres during the oncosphere host-infection process, i.e. whether the P2 protein gathers at the body surface as in *T. gondii* and *P. falciparum*, requires further study.

Due to the fact that *T. multiceps* infected goats will not show obvious clinical symptoms, it is difficult to detect in the early infective stage. This stage involves the adherence and migration of oncospheres in the major blood vessels of the intestine, and then, frequently, oncosphere transport to the nervous system including the brain and spinal cord via the circulatory system [46]. Detection of an antibody against coenurus could be useful for early detection and treatment of the infection. ELISA, DIGFA and Dot-ELISA diagnostic methods have been established for coenurosis [14–16]. However, due to the cross-reaction of natural antigens, previous studies have failed to establish a reliable diagnostic method. Although indirect ELISA based on a recombinant antigen against coenurus has been developed for diagnosis, it has deficiencies including low sensitivity [47]. In our study, indirect ELISA of recombinant *Tm*P2 was successfully established and optimized to detect coenurus in goats. The method had high sensitivity (95 %) and specificity (96.3 %) for 47 tested serum samples when compared with the results of necropsy. Moreover, there’s no cross reaction when using P2 protein for the detection of specificity of *E. granulosus*-positive sera. However, the indirect ELISA method against another protein (*Tm*GST) of *T. multiceps* established based on the same sera showed one cross reaction (1/8) (unpublished). Serodiagnosis through indirect ELISA was successfully used in experimental coenurus infection in sheep [48], however, seropositivity was observed only from the 35^th^ day p.i. In our experiment, the antibodies could be detected by indirect ELISA in the early stage of infection (3 weeks p.i), up to 17 weeks p.i.. The anti-*Tm*P2 serum of goats fell below the cut-off value about 2 weeks post-injection of praziquantel in the drug treatment group, and the antibody values remained below the critical value until the end of the experiment. Therefore, we conclude that indirect ELISA can be applied to the evaluation of coenurosis after the drug treatment. We did not detect the anti-*Tm*P2 antibody between 11 and 14 weeks p.i. in the control group; however, this phenomenon was not observed when we tested antibodies against another protein (*Tm*GST) of *T. multiceps* by indirect ELISA (data not shown). So, one can speculate that, in this period, due to decreased P2 expression, we cannot positively detect the anti-*Tm*P2 antibody. Although this is a potential weakness of the method, it can also be applied in clinic, because infected goats have shown obvious clinical symptoms between 11 and 14 weeks p.i.

## Conclusions

Recombinant *Tm*P2 is a suitable diagnostic antigen for coenurus infection. *Tm*P2-based indirect ELISA for detection of coenurus in hosts is sensitive and specific, and detects the parasite from only 3 weeks post-infection. The method will be useful for the diagnosis of coenurosis and for validating the effectiveness of drug treatment of infections.
